# Anesthetic Technique and Functional Outcomes in Modified Montgomery Thyroplasty

**DOI:** 10.3390/jpm13020194

**Published:** 2023-01-21

**Authors:** Manuel Granell, Ana Martín, Natsuki Oishi, Mar Gimeno Coret, Enrique Zapater

**Affiliations:** 1 Department of Anesthesia, Critical Care and Pain Medicine, Valencia University General Hospital, 46014 Valencia, Spain; 2 Faculty of Medicine, University of València, 46010 Valencia, Spain; 3 ENT Department, Valencia University General Hospital, 46014 Valencia, Spain

**Keywords:** vocal cord paralysis, modified Montgomery thyroplasty, laryngeal mask, general anesthesia, muscle relaxation, maximum phonation times and Voice Handicap Index-30

## Abstract

Background: Montgomery thyroplasty type I is a surgical technique indicated in vocal cord paralysis which consists of the paralyzed cord medialization, improving the voice quality. The objective of the study is to describe in detail the anesthetic method to obtain optimal post-medialization voice results. Methodology: Retrospective case series study including patients who underwent medialization thyroplasty using the modified Montgomery technique at the General University Hospital of Valencia between 2011 and 2021. The anesthetic technique consisted of general anesthesia with neuromuscular relaxation and a laryngeal mask. Pre- and post-surgical vocal functional data of maximum phonation times (MPT), G score, and Voice Handicap Index-30 (VHI-30) were evaluated. Results: All the patients presented an improvement in voice results, increasing MPT after surgery and a decrease in VHI-30 and G score postoperatively, with statistically significant differences between the pre- and post-surgical results (*p*-value < 0.05). There were no complications related to anesthesia or surgery. Conclusions: The use of general anesthesia with muscle relaxation in modified Montgomery thyroplasty may be a good option to consider. The use of a laryngeal mask for ventilation combined with a fiberoptic check allows direct visualization of the vocal cords intraoperatively, providing good functional voice results.

## 1. Introduction

**Figure 1:**
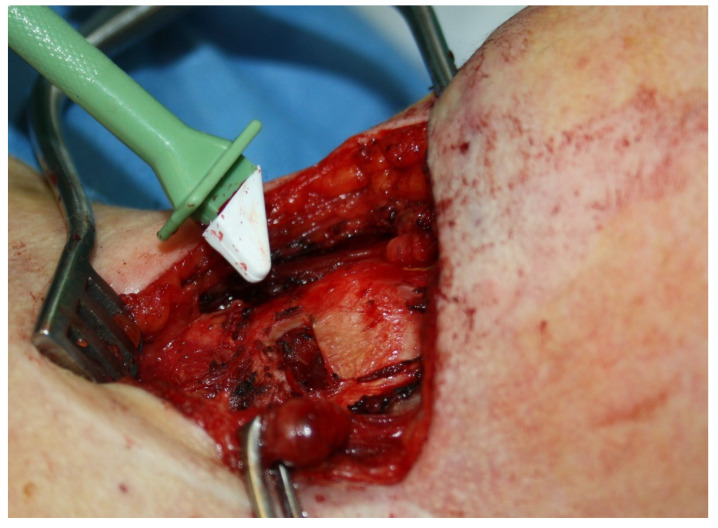
Meter for definitive prosthesis, inserted in the window created in the thyroid ala.

Montgomery medialization thyroplasty is a standardized and simplified procedure performed under local anesthesia and sedation indicated for unilateral paralysis of the vocal cord. The surgery consists of the medialization of the paralyzed cord thanks to the introduction of a prosthesis through a window in the thyroid cartilage, which improves glottic closure during speech, swallowing, and coughing. The classic Montgomery thyroplasty is based on the use of a silicone prosthesis whose size is chosen according to gender and intraoperative patient voice, which makes the procedure to be under local anesthesia and additional sedation. The sedation drugs described are midazolam (0.02–0.05 mg/kg); fentanyl (0.5–1 mg/kg); and increasing doses of 1% propofol, (0.3–0.5 mg/kg) until reaching the desired level of consciousness. The surgical procedure begins with a horizontal cervical incision followed by dissection by planes to expose the thyroid cartilage. The anatomical references will allow us to locate the window recommended by Montgomery to create a cartilaginous window to insert the silicone prosthesis [1]. Once the thyroid window is made, a meter is inserted to simulate the final prosthesis that will be used in the patient to medialize the cord (see Figure 1). During the voice test with the meter, the vocal cords can be observed with a flexible fiberscope to check the complete closure of the vocal cord. The surgeon tries different sizes (from number 6 to 13) and then asks the patient to speak. The prosthesis number is determined based on the patient’s voice during this test. Montgomery et al. recommend the use of local anesthesia and sedation because the surgical procedure is troublesome for the patient, particularly if a fibrolaryngoscope is used to monitor glottis changes [1,2].

Usually, it provides good functional results, however, there is a non-negligible percentage of suboptimal results. Suboptimal results are observed in 10% and the most common problem was the malposition or wrong size of the implants [3,4]. The ENT Department of General University Hospital of Valencia have years of experience in this surgical technique, they analyzed these unsatisfactory results due to the inadequate position of the Montgomery prosthesis and proposed two key points: the first is a surgical technique modification, and the second, an anesthetic method change. 

**Figure 2:**
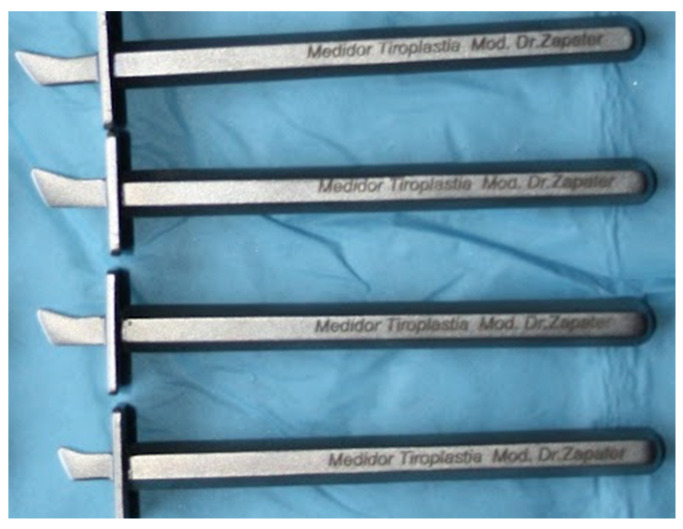
Different sizes of steel device designed by Zapater.

Our ENT surgeon developed a device designed specifically to locate more precisely where to place the prosthesis, based on individualized measurement according to each patient’s anatomy. Our surgeon individualizes the final location of the cartilaginous window, by means of a series of steel calipers (7 models, marked 6–12) designed by Zapater and manufactured by JMO (Barcelona, Spain), see Figure 2. They are narrower and thinner than those used in classic Montgomery thyroplasty (Boston Medical Products^®^). It is a modification of Montgomery´s surgical technique to improve the functional outcome [5]. 

In a study of series, in most cases it was necessary to modify the location of the Montgomery window; it was finally displaced inferiorly in 36.8% of the patients studied, and the displacement was inferoanterior in 31.6%. No modification of the theoretical window classically marked by Montgomery was required in only 5.3% of the cases [6]. Once the cartilaginous window is decided, the procedure does not differ from that proposed by Montgomery and the surgical instruments of Boston Medical Products^®^ are used, as well as the prosthesis adapted to the laryngeal size of each patient [1].

We perform the medialization thyroplasty under general anesthesia using a laryngeal mask. The classic Montgomery thyroplasty is based on the use of a silicone prosthesis whose size is chosen according to gender and intraoperative patient voice test, which makes the procedure to be under local anesthesia and additional sedation. However, this intraoperative voice test is not reliable as a predictor of final voice, based on our study of the effect of sedation on voice quality because the use of sedatives affects vocal parameters, therefore voice may not be a reliable measure in this surgery [7]. Some working groups already use general anesthesia and larynx mask that allows visualizing the endolarynx intraoperatively without impeding the medialization of the vocal cord and the rotation of the arytenoids, a situation that would occur with orotracheal intubation [8,9,10,11,12,13,14,15,16,17,18,19,20]. 

This work aims to describe in detail the anesthetic technique used in thyroplasty surgery, verifying the functional results after using this technique. Additionally, we will analyze the predictors of a good outcome in this surgical intervention. 

## 2. Materials and Method

This is a retrospective observational study, case series of the patients of the ENT Department of the General University Hospital of Valencia, diagnosed with unilateral cord paralysis and underwent medialization thyroplasty with Montgomery’s modified technique under general anesthesia. The study was conducted in accordance with the Declaration of Helsinki and approved by the Institutional Review Board of Valencia General University Hospital (Project reference 107/2022).

The variables studied statistically for the assessment of vocal function were: age at the time of surgery, gender, functional variables related to vocal cord paralysis such as cause of paralysis, Maximum phonation time (MPT), Voice Handicap Index-30 (VHI-30) [21], and GRBAS scale [22]. 

The MPT is the time that the patient can sustain a vowel. It is an objective test that evaluates glottic competence. This value is decreased in patients with unilateral vocal cord paralysis. The VHI-30 is a 30-item questionnaire that assesses the perception of the handicap of the patients with respect to the voice. The higher the score, the greater the perception of limitations in the quality of life. GRBAS scale is an assessment proposed by Hirano in 1981 that is based on the evaluation assessment made by the medical professional to determine the degree of dysphonia, hoarseness, breathiness, asthenia, and strain of a voice. This work focuses on evaluating the G of the GRBAS, which indicates the degree of dysphonia: severe, moderate, mild, or absent (evaluated from 3–0). 

The database was created in the Excel^®^ program (Office 2019, ©Microsoft 2021, version 16.54), which was imported into IBM^®^ SPSS^®^ Statistics (SPSS Inc., Chicago, IL, USA).

Statistical analysis was performed with the IBM^®^ statistical software program IBM^®^ SPSS^®^ Statistics (SPSS Inc., Chicago, IL, USA). The level of statistical significance was set at a *p*-value ˂ 0.05 and a confidence interval of 95%. We analyzed by descriptive statistics the epidemiological variables, as well as those related to vocal paralysis, and surgical variables, including mean and median for quantitative variables. For categorical variables, frequencies were calculated. 

The study of pre- and post-surgical variables was performed by paired samples Student’s *t*-test, including mean and median for quantitative variables. Student’s *t*-test for independent samples was used in the analysis of the remaining variables. In both cases, these tests were used in a normal distribution pattern. The G score did not follow a pattern of normality, the nonparametric Wilcoxon test was used for paired samples.

### Description of the Anesthetic Technique

Modified Montgomery thyroplasty was performed under general anesthesia with laryngeal mask insertion and muscle relaxation. The monitoring used was 5-lead ECG, non-invasive blood pressure, pulse oximetry (SpO_2_), capnography (EtCO2), depth of anesthesia using Physiometrix SEDLine^®,^ and neuromuscular relaxation using TOF-WATCH SX^®^.

**Figure 3:**
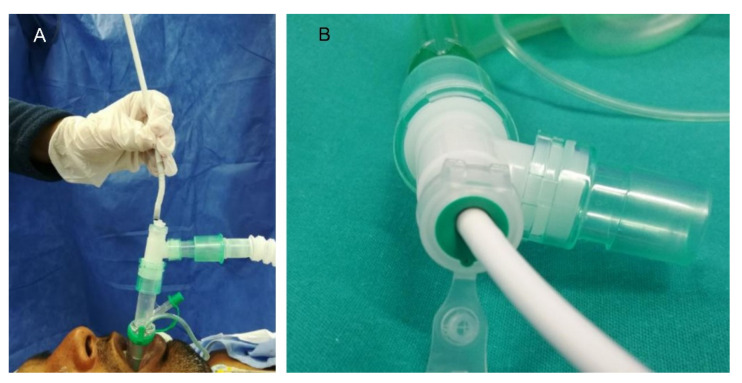
(**A**) Insertion of the flexible fiberscope through the ventilatory canal of the laryngeal mask for glottic visualization. (**B**) Proximal end of the laryngeal mask: T-connector. T-tube or connector allows for leak-free ventilation during fiberscope introduction.

**Figure 4:**
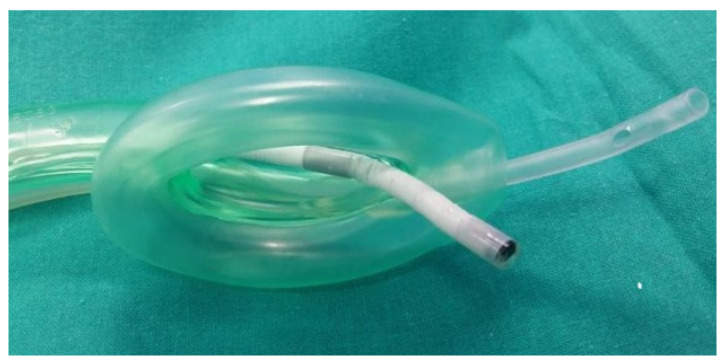
Distal end of the laryngeal mask airway. Exit of the fiberscope into the airway (white) and of the nasogastric tube into the esophagus (transparent).

Midazolam intravenous (0.03 mg kg^−1^) was administered to reduce the patient’s anxiety as premedication. At the time of anesthetic induction, it was administered intravenously fentanyl (0.7 mcg kg^−1^), propofol (2–2.5 mg kg^−1^), and rocuronium (0.6 mg kg^−1^). After 2 min in an optimal neuromuscular relaxation state (TOF ratio 0/0), Auragain laryngeal mask airway was introduced (see Figure 3A). A T-tube connector (Double Swivel Connector-Mallinchrodt™) was applied to the laryngeal mask (see Figure 3B) for subsequent insertion of the flexible fiberscope through it without compromising the anesthetized patient’s airway, ensuring adequate ventilation of the patient (Figure 3 and Figure 4). Repeated doses of rocuronium were administered (0.2 mg kg^−1^) during the surgery when the TOF ratio was above 2/4 to ensure the relaxation and lateralization of the healthy vocal cord, keeping the glottic lumen open and thus allowing different medialization measurements of the paralyzed vocal cord without putting the airway at risk.

Anesthetic maintenance throughout the sedative was performed with continuous infusions of IV propofol (6 mg kg h^−1^) and IV remifentanil (0.05–0.15 mcg kg min^−1^) according to patient needs. For laryngeal mask ventilation, 6–7 mL kg^−1^ tidal volume was delivered, with a respiratory rate adjusted to achieve an EtCO2 of 25–35 mmHg and a FiO^2^ of 21–50% (reducing to 21% when the electric scalpel was used to minimize the risk of ignition). Likewise, the peak pressure in the airways was evaluated at the time of the tests with the meters mentioned above. This pressure must not exceed 40 cmH_2_O, and it was necessary to check that the tidal volume was not reduced by more than 20% compared to the initial volume with the introduction of the definitive prosthesis meter. Therefore, monitoring the peak airway pressure at the time of prosthesis measurement influenced the decision on the definitive size of the final prosthesis.

After surgery, the patient was awakened: the intravenous infusions of propofol and remifentanil were stopped, and sugammadex (2–4 mg kg^−1^, according to the relaxation grade) was administered IV to reverse the effects of muscle relaxation and speed recovery [23]. Prior to the removal of the laryngeal mask, the recovery of reflexes and response to basic verbal commands was expected, as well as spontaneous ventilation by the patient with TOF ratio > 0.9.

Other medications administered were: a prophylactic dose of antibiotic, such as 1 g/100 mg of amoxicillin-clavulanate (1 g/100 mg) or ciprofloxacine (200 mg) IV 30 min before the surgery, metilprednisolone (1 mg kg^−1^) after the anesthesia induction, and paracetamol, ketoprofen and ondansetron iv 20 min before the surgery end.

## 3. Results

The study included 31 patients, 18 female, and 13 male. The mean age was 56.8 years (30–79 years). 

The use of general anesthesia with muscle relaxation with rocuronium in modified Montgomery thyroplasty allows greater safety in the manipulation of the larynx during surgery by facilitating the passage of airflow through the airway. Intraoperatively and during the recovery, there were no anesthetic or surgical complications in any patient. The use of a laryngeal mask for ventilation combined with a fiberoptic check allows direct visualization of the vocal cords intraoperatively without compromising the airway or obstructing the view of the vocal cords during surgery. No ventilatory complications were observed. 

The causes of vocal cord paralysis studied were: paraganglioma (vagal, carotid, and/or jugular) surgery, cervical herniated discs surgery, acoustic neurinoma surgery, cervical schwannoma surgery, esophageal cancer surgery, thyroid or parathyroid surgery, thoracic surgery of aortic aneurysms, and idiopathic paralysis of the vocal cord.

On the other hand, all functional results improved (regarding the variables under study, their descriptive statistics are shown in Table 1, Table 2 and Table 3).

**Figure 5:**
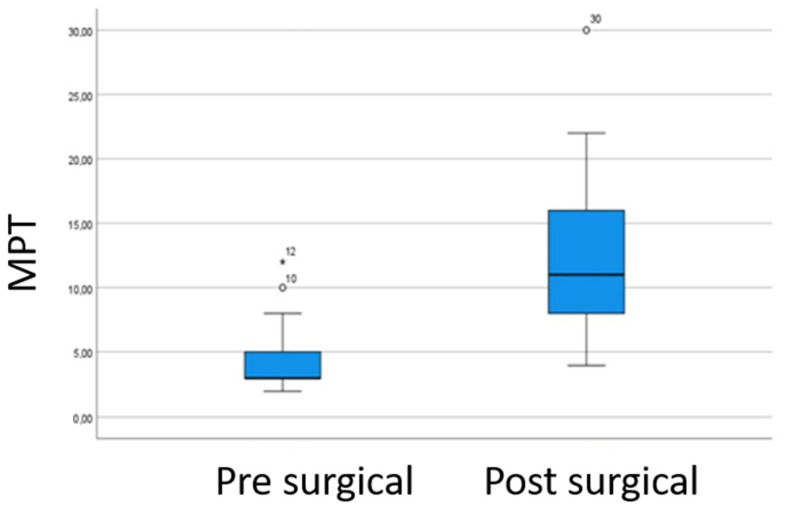
Comparison of the pre- and post-surgical MPT values. The post-surgical MPT values were higher than the pre-surgical, indicating improvement in the glottal closure.

The post-surgical MPT values were higher than the pre-surgical, indicating improvement in the glottal closure. The mean absolute increase in maximum phonation time was 8.5 s. Figure 5. There were statistically significant differences in *p*-value < 0.05 (*p*-value = 0.000) indicating that the MPT varies after the surgery and is no statistically significant correlation between variables.

**Figure 6:**
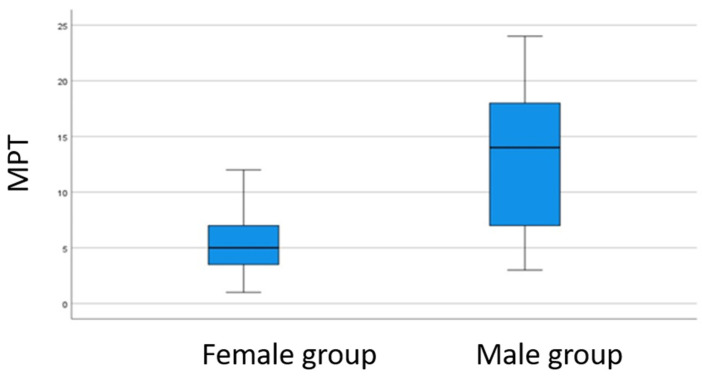
Comparison of the MPT values between gender groups. A significantly higher increase difference is observed in the male group.

In the study of gender and the absolute increase in the MPT, the Student’s *t*-test of independence showed a *p*-value < 0.05 (*p*-value = 0.000) indicating there were differences between both sexes. The linear relationship between these variables, the Pearson correlation coefficient R = 0.618, with a *p*-value < 0.05 (*p*-value = 0.000) was observed, establishing a greater increase in the maximum phonation time in males, see Figure 6.

**Figure 7:**
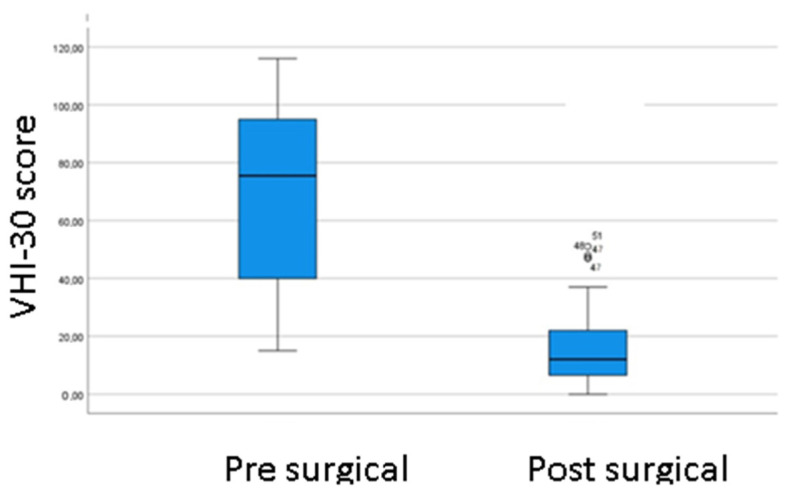
Comparison of the pre- and post-surgical VHI-30 score.

The Voice Handicap Index-30 before and after surgery result: an improvement in the perception of voice-related disability was observed in all patients. There are statistically significant differences between the means, *p*-value < 0.05 (*p*-value = 0.000), Figure 7.

In the study of the correlation between the VHI-30 pre- and post-surgical questionnaires, there is no evidence of a statistically linear correlation between them, with a *p*-value> 0.05 (*p*-value = 0.057), no can be inferred that they change together in a linear fashion.

**Figure 8:**
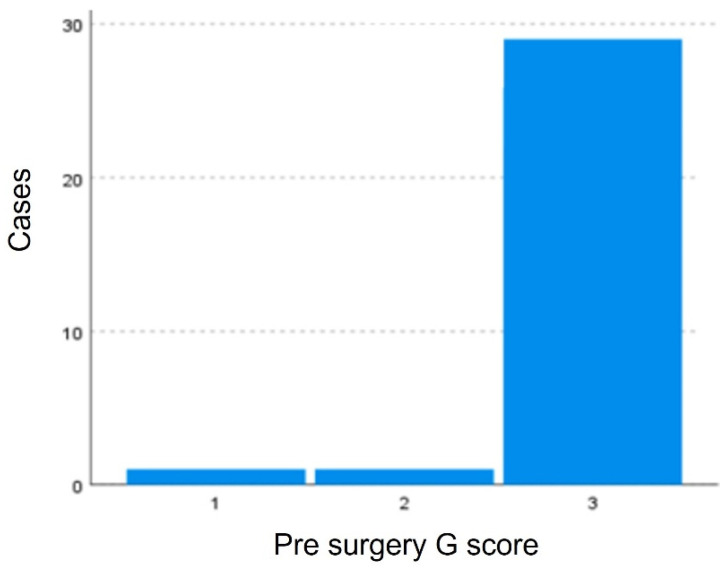
Distribution of patients according to the G scale. Greater clustering of pre-surgical patients with high degrees of dysphonia (G3) is observed.

**Figure 9:**
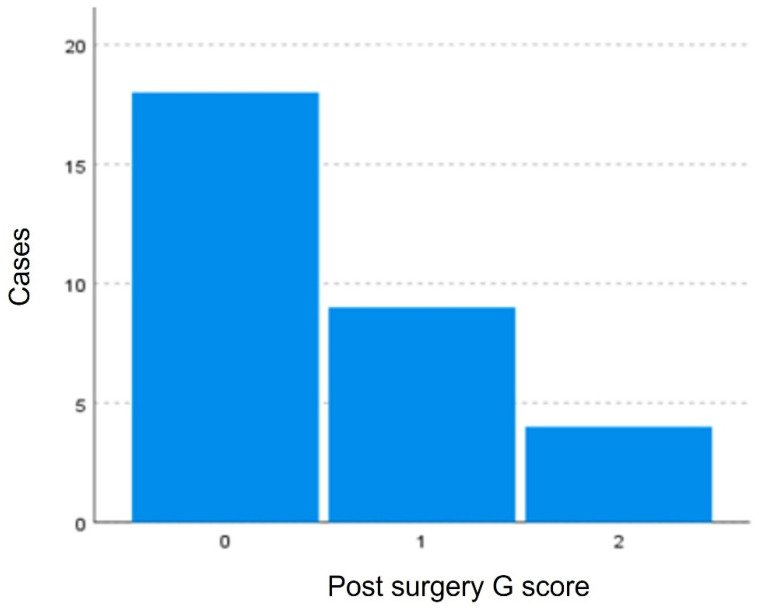
Distribution of patients according to the G scale. More patients with fewer dysphonia cases were found: G0, G1, G2, and no cases of G3.

Variable G did not pass the normality tests after logarithmic transformation, the nonparametric Wilcoxon test for paired samples was used. Statistically significant differences with a *p*-value < 0.05 (*p*-value = 0.000) exist between the degree of dysphonia pre-surgery and post-surgery indicating that the surgery modified the G score. This difference is evident in Figure 8 and Figure 9, where a greater clustering of pre-surgical patients with high degrees of dysphonia (G3). In the post-surgical evaluation, a greater number of fewer dysphonia cases were found (G0–G2).

## 4. Discussion

Montgomery thyroplasty is one of the most used surgical solutions for vocal cord paralysis, it provides a systematic and easy-to-reproduce technique. This work focuses on how we do it to mitigate the differences in the results of the procedure by individualizing it to each patient because one of the objectives of this work is the description of the anesthetic technique used in the modified Montgomery thyroplasty, fundamental for the correct result of this intervention. Surgical innovation is the use of a device to increase precision in an individualized way for the implantation of the optimal size of the prosthesis in the appropriate place. The anesthetic method with laryngeal mask and muscle relaxants, as a special novelty with respect to literature, allows the visualization of the endolaryngeal effect of the prosthesis which facilitates the choice of the appropriate size of the prosthesis ensuring optimal ventilation. The limitations of this study are that it is a single-center and a retrospective study with a small sample size, however, the number of patients included is comparable to published series of studies on thyroplasty.

In the classic Montgomery thyroplasty, local anesthesia with sedation is used during the surgery. This method requires waking up the patient and requesting their phonation, relying on the monitoring of the patient’s intraoperative voice to choose the size and type of prosthesis [1]. However, we find the following disadvantages: sedative drugs can alter voice quality despite individualized sedation, there is an obvious difficulty in achieving a balance between the optimal level of sedation and the strict control of the airway, is uncomfortable in most cases, and also the feeling of shortness of breath and coughing fits are frequent at the time of medialization. On the other hand, general anesthesia during thyroplasty surgery would allow us to objectively monitor medialization by video-laryngoscopy, better airway control, and greater comfort, as well as greater precision when choosing and placing the prosthesis. Nowadays, some work groups already use general anesthesia with a laryngeal mask and fiberscope [8,9,10,11,12,13,14,15,16,17,18,19,20], but our work group goes one step further and proposes neuromuscular relaxation during general anesthesia to keep the healthy cord sufficiently abduced so as not to affect the medialization of the cord that we want to medialized. Rocuronium bromide was the neuromuscular relaxant of choice. It is a non-depolarizing blocker with a steroidal structure, whose action is completely antagonized by its antidote, Sugammadex, with a rapid onset time (2 min at standard intubation doses or 1 min in rapid intubation sequences) with an intermediate duration of action. according to the dose. It was marketed in the United States in 1995 and in Chile in 1996 under the trade name Esmerón^®^. Its mechanism of action lies in competition for nicotinic cholinergic receptors at the motor end plate. It is used as an adjunct to general anesthesia to facilitate tracheal intubation and rapid sequence induction, as well as to relax skeletal muscles in surgery. It is also indicated as an adjuvant in the ICU to facilitate tracheal intubation and mechanical ventilation. It is indicated in pediatric patients older than 1 month and adults. It is administered intravenously at an induction dose of 0.6–1.2 mg/kg. The maintenance dose is 0.1–0.2 mg/kg in boluses as needed or 5–15 mg/kg/min if administered by continuous infusion. It is eliminated mainly through the bile, and urinary (in unchanged form). Sugammadex is indicated for the reversal of neuromuscular blockade induced by rocuronium or vecuronium and is indicated for the routine reversal of rocuronium-induced blockade. The recommended dose of sugammadex depends on the level of neuromuscular blockade to be reversed.

As seen in this work, muscle relaxation does not cause any complications and ensures the passage of the air column sent by the ventilator during surgery [23]. Bearing in mind that the surgical procedure is based on intraoperative manipulation of the glottis, it is important to guarantee correct ventilation of the patient at all times. In addition, the need to maintain muscle relaxation during surgery becomes important, increasing anesthetic safety and reducing the possibility of complications due to laryngospasm when manipulating the airway during surgery [24,25].

On the other hand, the intraoperative control of the voice through the patient’s phonation is one of the pillars on which numerous authors are based, including Isshiki and Montgomery [1,2,26] for the final choice of the prosthesis to be used. However, our team concluded that intraoperative voice quality with the use of sedation is not a reliable parameter [7]. A comparative study was carried out between the voice without sedation and the effects of sedation. A subjective and objective study by acoustic analysis revealed the changes. The subjective perceptual assessment of speech in patients under sedation showed an ataxic dysarthria-like speech characterized by bradylalia, low pitch, and an utterance pattern. All patients in this series demonstrated monotonous and weak voices, poor prosody, and irregular and slow rates of speech. Sedation drugs influenced the voice quality in patients under sedation. Observed changes in the objective analysis parameters included higher values for jitter local and shimmer local and lower for pitch and Harmonic Noise Ratio in patients under sedation. Therefore, voice evaluation during the thyroplasty procedure may be compromised in sedated patients. General anesthesia could be an alternative, focusing our attention on monitoring the glottis with a fibrolaryngoscope during the surgical procedure [7,11].

This anesthetic alternative uses the laryngeal mask with a T-connector, which allows the insertion of a flexible fiberoptic laryngoscope without compromising the airway, providing direct visualization of the vocal cords during the insertion of the sizers and prostheses. The choice is made based on the medialization and the peak pressure in the airway at the introduction of the meter, which must be less than 40 mmHg so as not to affect ventilation during surgery. Therefore, endoscopic control during surgery is one of the key elements of the surgical technique and the decision of the prosthetic size.

Satisfactory functional results were obtained after surgery, and an improvement in the MPT of 8.52 s was achieved, comparable to the results with the classic Montgomery technique of mean MPT of 8.32 s [1,2,27] and of another study carried out in our hospital by Zapater et al. [5].

This study also shows statistically significant differences (*p*-value < 0.05) between men and women in the result. Men improve more than women after undergoing modified Montgomery thyroplasty. Montgomery also obtained similar results in his study, where an average increase in MPT of 11 s was obtained in men, while in women the average increase was 5.63 s [1]. There are authors who affirm that the discrepancy in the outcome is due to the morphology of the laryngeal framework: men have a smaller angle between both thyroid plates than women, and the smaller the angle, the greater the increase in the maximum post-surgical phonation time [28,29]. 

The significant decrease in the VHI-30 questionnaire score after surgery (*p*-value < 0.05) in our series is a good indicator of surgery success, however, it cannot be affirmed that having a high VHI prior to surgery conditions a certain outcome after surgery. This post-surgical score is maintained years after the intervention reflecting the stability of the functional result of medialization thyroplasty [30]. 

In this procedure cooperation between the surgeon and anesthesiologist is essential. The functional results are satisfactory and have been included in the study because they reflect the benefit of the multidisciplinary approach.

## 5. Conclusions

The use of general anesthesia with muscle relaxation in modified Montgomery thyroplasty may be an option to consider compared to the use of local anesthesia with sedation used in classic Montgomery thyroplasty, avoiding the inconveniences of local anesthesia and the effects and the effects of sedatives on the voice. Muscle relaxation with rocuronium allows greater safety in the manipulation of the larynx during surgery by facilitating the passage of airflow through the airway.

The use of a laryngeal mask for ventilation combined with a fiberoptic check allows direct visualization of the vocal cords intraoperatively without compromising the airway or obstructing the view of the vocal cords during surgery. 

The functional results obtained by studying the maximum phonation time, G score, and the Voice Handicap Index-30 have shown clinical improvement after the intervention, especially in male patients.

## Figures and Tables

**Table 1:** Comparison of maximum phonation time MPT (seconds) pre and post-modified Montgomery thyroplasty.

	MPT Pre	MPT Post	MPT Improvement
Media	4.3	13.7	9.4
Minimum	2	4	1
Maximum	12	30	24

**Table 2:** Comparison of VHI-30 scores pre and post-modified Montgomery thyroplasty.

	VHI-30 Pre	VHI-30 Post
Media	72	14
Minimum	15	11
Maximum	116	92

**Table 3:** Comparison of G scores pre and post-modified Montgomery thyroplasty.

	G Score Pre	G Score Post
Media	2.90	0.55
Minimum	1	0
Maximum	3	2

## Data Availability

Not applicable to this article as no new data were created or analyzed in this study.
